# Impacts of pseudomonas fluorescent bacterial fertilizer addition on the soil environment and fruit yield under water stress in greenhouse grape

**DOI:** 10.3389/fmicb.2025.1540628

**Published:** 2025-02-06

**Authors:** Yanting Gao, Hongjuan Zhang, Rui Zhang, Zhen Huang, Changyu Yang

**Affiliations:** College of Water Conservancy and Hydropower Engineering, Gansu Agricultural University, Lanzhou, China

**Keywords:** microbial community, bacterial fertilizer, enzyme activity, water stress, greenhouse grape

## Abstract

Bacterial fertilizers, which contain beneficial soil microorganisms, are becoming more widely used as they can mitigate the problems of crop yields reduction and soil environment degradation caused by the overuse of chemical fertilizer. However, the impact of bacterial fertilizer on greenhouse grape yields and the rhizosphere soil environment has not been assessed in arid and semi-arid region of Northwest China. Thus, a 2-year field trial was conducted with five treatments: adequate water supply without bacterial fertilizer (CK); mild (W1), moderate (W2) water stress and small (F1), maximize (F2) fertilizer cross-combination, respectively. The results indicated that water stress had a negative impact on the accumulation of dissolved organic carbon (DOC), microbial biomass carbon (MBC), and microbial biomass nitrogen (MBN) in the rhizosphere soil. The addition of pseudomonas fluorescent bacterial fertilizer significantly increased the content of available phosphorus (AP), DOC, MBC and MBN content. The W1F2 treatment significantly increased the activities of urease, catalase and sucrase (*p* < 0.05). The W1F1 and W1F2 treatments increased fungal and bacterial diversity. Bacterial community composition was closely related to soil total organic carbon (TOC), soil organic matter (SOM), total nitrogen (TN), total phosphorus (TP), MBC, and sucrase, while fungi community composition was significantly related to Nitrate-N (NO_3_^−^-N), TN, and sucrase. Additionally, compared with CK treatment the yield and economic benefit of the W1F2 treatment increased by 35.44 and 44.04%, respectively. Therefore, W1F2 is recommended as the optimal water and fertilizer management scheme for efficient greenhouse grape production in the arid and semi-arid region of Northwest China.

## 1 Introduction

In the context of global climate change, water resources have become an important constraint on sustainable socio-economic development worldwide (Benlloch-González et al., 2015). Studies have shown that water stress hinders various physiological processes, such as photosynthesis, respiration, ion uptake, and nutrient metabolism in plants, which ultimately leads to developmental retardation and growth reduction (Bhandari et al., 2023; Hancioglu et al., 2021). Reduced growth and suboptimal yields due to water limitations and nutrient deficiencies are particularly severe in arid and semi-arid region (Ortiz-Álvarez et al., 2023; Wahab et al., 2022). Therefore, improving the soil environment under water stress conditions in crops is essential to promote crop growth and mitigate yield losses (Seleiman et al., 2021).

Grape (*Vitis vinifera* L.) is one of the most important fruit and cash crop worldwide. Research has shown that grapes possess a variety of health benefits, such as antioxidant (Liu et al., 2018), antidiabetic (Irak et al., 2018), anti-inflammatory (Barona et al., 2012) gut microbiota regulator (Han et al., 2020), anti-cancer (El-Din et al., 2019), and cardioprotective effects (Svezia et al., 2020). Grapes are not only consumed as fresh fruit, but are also an indispensable raw material for producing various products such as wine, grape juice, and raisins, along with their by-products that have multiple uses in the food industry. However, average yields differ greatly among grape-growing countries (Zhou et al., 2022). In order to obtain high yields, traditional agriculture relies on large amounts of chemical fertilizer inputs. However, excessive application of chemical fertilizers not only creates a series of negative environmental problems, but also reduces soil quality, such as exacerbating the decline in soil organic matter and fertility, accelerating soil acidification, and thus reducing crop yields (Qiao et al., 2018). The delayed cultivation grape was a table grape cultivation mode that is very suitable for the climate characteristics of the cold and cool regions in Northwest China, which used the shading facilities of the solar greenhouse to extend the dormancy period of grapes, thus delaying the ripening time of grapes, and making them mature and available in the winter, so as to increase the price, as well as quality of grapes, and has played an important role in the annual balanced supply of table grapes and the increase of the income of the local farmers. Therefore, exploring environmental friendly green fertilizer to reduce the environmental problems associated with chemically synthesized fertilizer and to achieve agriculture sustainability has received increasing attention from researchers.

Soil consists of three main components: minerals, organic matter, and microorganisms. Among them, microorganisms, including bacteria and fungi, directly or indirectly affect soil quality and crop yields (Wan et al., 2024) through pathways such as accelerating soil organic matter (SOM) turnover and increasing nutrient mineralization rates (Yang et al., 2022). Additionally, microbial activity is crucial for plant root nutrition because beneficial soil microorganisms are directly involved in formation of soil fertility, including matter and energy conversion, humus formation and decomposition, nutrient release, and nitrogen fixation (Zhang et al., 2022). Microbial activities in the rhizosphere, especially Acropora Mycorrhizal Fungi (AMF) and Plant Growth Promoting Rhizobacteria (PGPR), can assist plants in water uptake (Ouhaddou et al., 2022), thereby improving plant survival under water stress (Tardieu, 2022). Therefore, soil microorganisms are recognized as key mediators of nutrient cycling, with their abundance and diversity become important indicators for soil health assessment (Delgado-Baquerizo et al., 2020). However, the composition and diversity of soil microorganisms are influenced by various factors, such as soil texture, SOM content, pH, nutrient availability and soil microbial activity (Linton et al., 2020), making it difficult to achieve artificially targeted regulation of the microbiota in the ecosystems in their natural state. Microbial fertilizer has been more and more widely used in many countries because they contain a large number of specific beneficial microorganisms, which form a structured soil microbial community through antagonism, competition, and symbiosis with the original microorganisms in the soil, which can play an important role in maintaining and improving soil fertility (Meddich, 2023), improving soil structure (Anli et al., 2022), promoting plant growth and development (Dimkić et al., 2022), enhancing disease resistance (Ait Rahou et al., 2022), and reducing the risk of soil pollution (Sevilla-Perea and Mingorance, 2015; Yang et al., 2020) and so on. The application of microbial fertilizer to enhance the abundance and overall activity of soil microorganisms by “artificially” increasing the amount of beneficial microorganisms not only improves soil microbial diversity, but also alters the functional diversity of soil microorganisms (Chen et al., 2014). Studies have demonstrated that antagonistic antimicrobial fertilizers could prevent and control cucumber soil-borne blight, reduce the number of pathogenic bacteria in rhizosphere soil, and improve rhizosphere soil microflora (Li et al., 2016). Moreover, adding microbial fertilizer has been shown to promote corn growth, increase yields, and potentially replace 10–30% of compound chemical fertilizers (Liu et al., 2022). It was confirmed that arbuscular mycorrhizal colonization can mitigate the deleterious effect of water stress on growth and flower yield of the snapdragon ornamental plant (Asrar et al., 2012), and the improved growth conditions of Aurelia roots were attributed to the higher WUE in the inoculated roots after mycorrhizal symbiosis (Bahador et al., 2023). The reduction in photosynthetic pigments such as chlorophyll is one of the adverse effects of drought stress (Hussain et al., 2019), while severe decline in root growth can greatly reduce nutrient uptake by the soil. However, the application of biological and organic fertilizer under water deficit conditions can mitigate the adverse effects of water stress by increasing leaf chlorophyll synthesis and concentration through the provision of nitrogen, production of growth enhancers, increase in microbial populations, and enhancement of nutrient availability and uptake, which in turn affects the physiological traits of the plant (e.g., photosynthetic pigments and nutrient uptake) to mitigate the adverse effects (Lalitha and Santhakumari, 2021; Zamani et al., 2023). Therefore, the application of beneficial microbial fertilizer is one of the effective ways to achieve sustainable agriculture by promoting the development of novel microbial flora within root system of crops, thereby improving the soil environment, which both to alleviate water stress and promote the growth and development of plants and mitigate the adverse effects on the environment (Pineda et al., 2017; Karimzadeh Asl and Hatami, 2019).

In this study, the rhizosphere soil of “Red Earth” greenhouse grapes in arid areas was studied, and different fertilizer treatments under water stress were established. We analyzed yield, soil physicochemical properties, enzyme activities and soil microbial composition to provide a theoretical basis for the scientific water conservation and fertilization of greenhouse grape in arid and semi-arid region. The objectives of this study were to: (1) analyze the effects of bacterial fertilizer addition under water stress on the physicochemical properties, enzyme activities of greenhouse grape rhizosphere soil; (2) reveal the bacterial and fungal community composition and how it responds to fluorescent pseudomonas bacterial fertilizer; and (3) screen for the most effective fertilizer addition amounts to improve the rhizosphere soil environment of greenhouse grape under water stress conditions. We hypothesized that there is a certain correlation between soil properties, enzyme activities and microbial composition, and can improve soil nutrients and promote soil enzyme activities, and the addition of fluorescent pseudomonas bacterial fertilizer affect the diversity, community structure and composition of soil microorganisms. This is helpful to further explore the mechanism or principle of the effect of different addition amounts on yield.

## 2 Materials and methods

### 2.1 Experimental cite description

**Figure 1:**
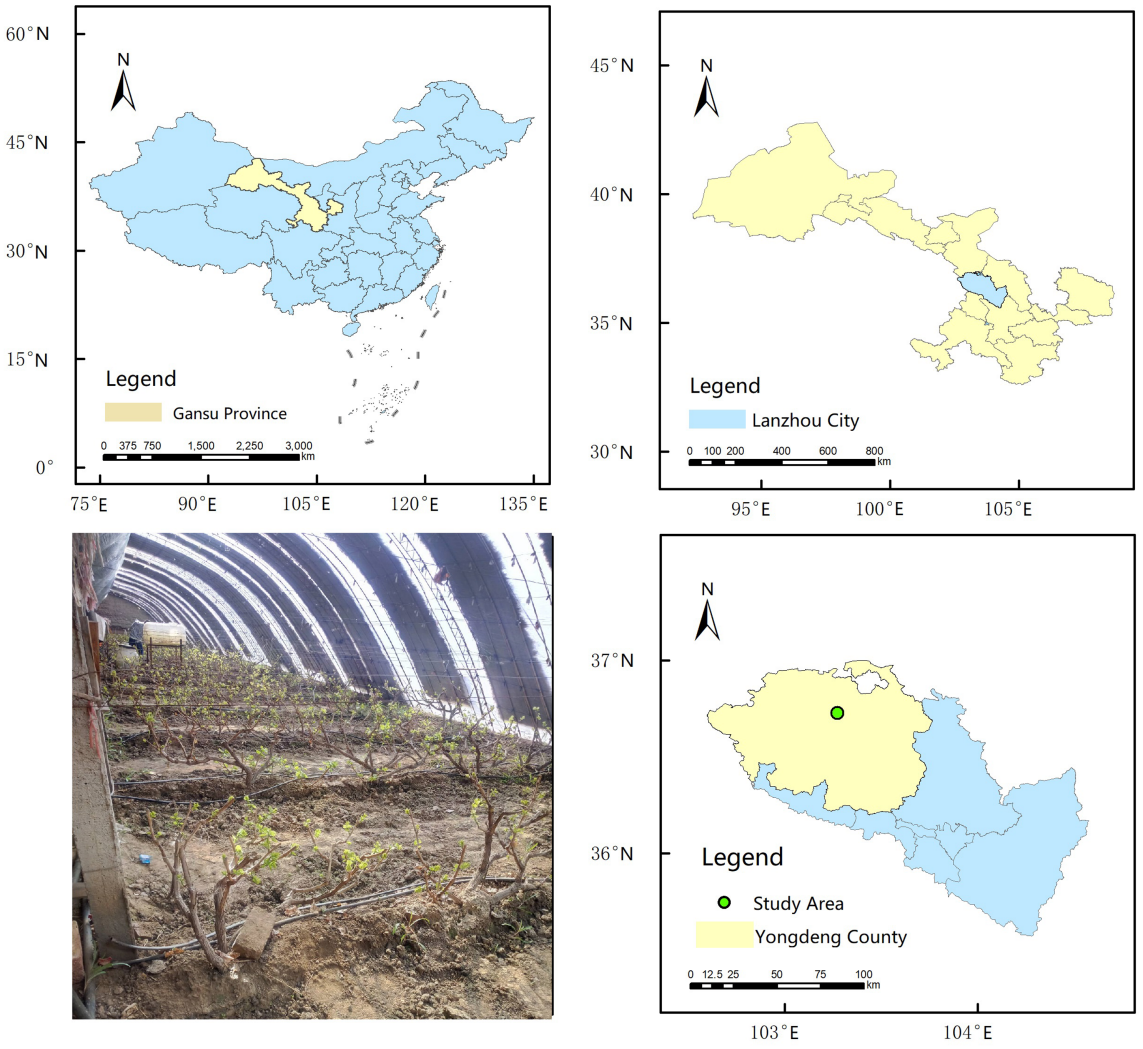
Location of the experimental site.

The field experiment was conducted from 2019 to 2020 at the Facility Viticulture Test Base (36°43′34″N; 103°16′24″E; altitude: 2100 m) (Figure 1) in Yongdeng County, Lanzhou City, Gansu Province, China. The test base is located in a semi-arid region with a typical continental monsoon climate with an annual average rainfall, evapotranspiration, and temperature of 290 mm, 1,000 mm, and 5.9°C, respectively. The experimental field soil is classified as loam, with the following initial properties: volumetric soil moisture content (θ_f_) 29.2%, density 1.42 g cm^−3^, and pH 8.15.

### 2.2 Experimental design and management

“*Red globe*,” a 5-year-old Eurasian grape variety, was used as the test material, which was planted in a plastic greenhouse of 8 m × 80 m with an earth wall straw curtain. The rows of grape were orientated perpendicular to the longitudinal direction of the greenhouse. Each row (each treatment) contained eight grapes with a row spacing 2.0 m and the plant spacing 0.8 m. The cultivation method of a single-arm Y-shaped low single-hedge frame was adopted.

The growing period of grapes were divided into five stages in the light of local protected-cultivation grape water consumption and irrigation experience: germination stage (May 15–May 24), shoot elongation stage (May 25–June 22), flowering stage (June 23–July 15), fruit enlargement stage (July 16–September 14), and coloring maturity stage (September 15–October 18). Three treatments of auxiliary experiment were set up to investigate the effects of different water regulation on soil physicochemical properties and enzyme activities in greenhouse grape during the growing season in 2019 (Table 1): W0 (CK) treatment (the control treatment) was adequate water supply (soil moisture content was (75% ~ 100%) θ_f_); W1 treatment was mild water stress (soil moisture content was (65% ~ 90%) θ_f_); W2 treatment was moderate water stress (soil moisture content was (55% ~ 80%) θ_f_). In order to further study the effects of adding bacterial fertilizer under different water regulation on the rhizosphere soil environment of grape in the greenhouse, five treatments of main experiment were set up in 2020 (Table 1): W0F0 (CK) treatment (the control treatment) was adequate water supply and no fertilization (soil moisture content was (75% ~ 100%) θ_f_); W1F1 treatment was mild water stress and small fertilizer (soil moisture content was (65% ~ 90%) θ_f_ and adding 60 kg hm^−2^ of bacterial fertilizer); W1F2 treatment was mild water stress and maximize fertilizer (soil moisture content was (65% ~ 90%) θ_f_ and adding 120 kg hm^−2^ of bacterial fertilizer); W2F1 treatment was moderate water stress and small fertilizer (soil moisture content was (55% ~ 80%) θ_f_ and adding 60 kg hm^−2^ of bacterial fertilizer); W2F2 treatment was moderate water stress and maximize fertilizer (soil moisture content was (55% ~ 80%) θ_f_ and adding 120 kg hm^−2^ of bacterial fertilizer). The experimental plots in both years were in a randomized block design with three replications for each treatment and plot size of 8 m × 2 m.

**Table 1:** The auxiliary field experiment in 2019 and the main experiment design in 2020 for greenhouse grape.

Year	Treatment	Irrigation treatment	Lower and upper limit of soil moisture content in different treatments (%θ_f_)	Fertilizer treatment	Fertilizer amount (kg hm^−2^)
2019	W0 (CK)	W0 (adequate supply)	75% ~ 100	No fertilizer	0
	W1	W1 (mild stress)	65% ~ 90	No fertilizer	0
	W2	W2 (moderate stress)	55% ~ 80	No fertilizer	0
2020	W0F0 (CK)	W0 (adequate supply)	75% ~ 100	F0 (no fertilizer)	0
	W1F1	W1 (mild stress)	65% ~ 90	F1 (small fertilizer)	60
	W1F2	W1 (mild stress)	65% ~ 90	F2 (maximize fertilizer)	120
	W2F1	W2 (moderate stress)	55% ~ 80	F1 (small fertilizer)	60
	W2F2	W2 (moderate stress)	55% ~ 80	F2 (maximize fertilizer)	120

θ_f_ was the volumetric soil moisture content, which value taken in this study was 29.2%.

A drip irrigation system with a “one pipe per row” configuration was used in the greenhouse grape field trial, with emitters delivering water at a rate of 3 L h^−1^. The irrigation amount and irrigation time were determined by the soil moisture content and measured using the water meter which was installed in each plot along with the valves. When the soil moisture in the field reached the lower limit of the experimental design, the irrigation was performed and stopped at the upper limit. The soil moisture ratio was maintained at 0.5, with a planned wetting depth of 80 cm. To monitor and maintain water stress levels in the greenhouse, soil water content was measured by the drying method, with soil samples taken every 7 d throughout the reproductive period and additional measurements taken before and after irrigation when soil water content approached the lower limit of irrigation control (Table 1), and a polyethylene geomembrane with a thickness of 2 mm was laid between the plots (to the soil depth of 1 m) to prevent the diffusion of soil water between different plots.

*Pseudomonas fluorescens* had the ability to antagonize many plants pathogenic fungi and was the dominant phosphorus bacterium in the soil. Pseudomonas fluorescent bacterial fertilizer was chosen in the experiment for its broad-spectrum bacteriostatic and growth promoting effects, which was purchased from Shanghai Ruichu Biotechnology Co., Ltd.^1^ with the brand REBIO, and the product number Y0024. The composition of the ingredients per kg of bacterial fertilizer was as follows: 55 g of fluorescent *pseudomonas* solution (1 g of fluorescent *pseudomonas* solution contains ≥10 billion live bacteria), 110 g of corn flour, 110 g of soybean cake powder, 73 g of bran powder, 73 g of K_2_HPO_4_, 29 g of KH_2_PO_4_, 3 g of MgSO_4_, and 547 g of peat. To ensure uniformity of fertilizer application, *Pseudomonas fluorescens* was mixed with sand at a mass ratio of 1:50, and then applied in three separate applications approximately 20 cm around the roots of each grape plant in each plot at the beginning of germination period (May 15th), the flowering period (June 16th) and the fruit expansion period (August 16th), respectively. Pruning, fertilization, pest and disease control, weeding and other field management practices for each plot were uniformly carried out in accordance with local viticultural management experience, among which the fertilization measure was as follows: on February 24th, basal fertilizer (chicken manure; 5,000 kg hm^−2^) was applied along with 2 kg diammonium phosphate and 4 kg ammonium bicarbonate; On June 16th, each treatment received 1 kg diammonium phosphate, 0.8 kg calcium ammonium nitrate for agriculture, 0.8 kg of organic fertilizer, and 0.5 kg of potassium magnesium sulfate, respectively; On August 16th, 0.8 kg diammonium phosphate, 0.8 kg calcium ammonium nitrate for agriculture, 0.8 kg organic fertilizer, and 0.6 kg potassium and magnesium sulfate were applied.

### 2.3 Grape yield, water use efficiency and economic benefit measurement

#### 2.3.1 Yield

During the grapes harvest, each plot (treatment) was harvested individually and measured for yield using the electronic balance (GB/T7722-2005) with an accuracy of 0.01 kg.

#### 2.3.2 Water use efficiency and irrigation water use efficiency

The formulas for WUE, and IWUE are as follows, respectively:


(1) WUE=Y/W

(2) IWUE=Y/I

Where *Y* is the economic yield, kg hm^−2^; *W* is the total water consumption during the whole growth period of grapes, m^3^ hm^−2^; *I* is the total irrigation water amount during the whole growth period of grapes, m^3^ hm^−2^.

#### 2.3.3 Economic benefit

The EB was calculated using the following equation:


(3) EB=P−TC

Where *P* is profit from grape yield (CNY hm^−2^), and TC (CNY hm^−2^) is the total cost which consisted of irrigation costs, fertilizer costs and labor costs.

### 2.4 Soil sample collection and property measurement

Soil samples were collected during different grape growth periods in both 2019 (May 19th, June 18th, July 15th, August 20th, and October 20th) and 2020 (May 15th, June 15th, July 20th, August 15th, and October 15th). The surface litter and impurities were moved away, and then soil samples (depth of 20–40 cm) of 10 cm away from grape roots from three random regions of the plot were collected by using a core sampler (20 mm internal diameter). After the root system and other impurities were picked out, the soil samples of the same treatments were thoroughly mixed and sieved (2 mm mesh), and then divided into three parts, of which one part was stored at 4°C for analysis of enzyme activity, one part was frozen in refrigerator of −20°C for the extraction of soil macrogenomic DNA, and the third part were naturally air dried for the determination of physicochemical properties. The measurement of each index was completed within 1 month after sampling was completed.

Total nitrogen (TN), nitrate nitrogen (NO_3_^−^-N), and ammonium nitrogen (NH_4_^+^-N) contents were measured by the Kjeldahl method (Boell and Shen, 1954). Soil organic matter (SOM) was determined by the potassium dichromate external heating method (Wang et al., 2023). Total phosphorus (TP) and available phosphorus (AP) were analyzed by a UV2450 ultraviolet spectrophotometer (Shimadzu, Japan) (Li et al., 2022). The soil total organic carbon (TOC) content was measured using a carbon and nitrogen combined analyzer (Multi N/C 2100 s, Jena, Germany) after removing inorganic carbon with 0.5 mol L^−1^ dilute hydrochloric acid. The dissolved organic carbon (DOC) content was determined with a carbon and nitrogen combined analyzer (Multi C/N 2100 s) after leaching with ultrapure water (water: soil = 5: 1). The microbial biomass nitrogen (MBN) and microbial biomass carbon (MBC) content were determined using a carbon and nitrogen combined analyzer after 0.5 mol L^−1^ K_2_SO_4_ extraction (Multi C/N 2100 s) (Xu et al., 2016).

Soil urease activity was determined by the Kits of Art. No. G0301F (Suzhou Gerisi Biological Technology Co. LTD) through spectroscopic method. To determine the sucrase activity, 5 g of air-dried soils were incubated for 24 h at 37°C with 15 mL of 8% sucrose, 5 mL of phosphate buffer at pH 5.5, and 0.1 mL of toluene. The glucose released by sucrase reacted with 3-5-dinitrosalicylic acid and then was measured based on the absorbance at 508 nm (UV-2450, Shimadzu Corporation, Kyoto, Japan) (Wu et al., 2020). The results were expressed as mg glucose g^−1^ h^−1^. Soil catalase activity was determined by the KMnO_4_ liquid titration method and expressed as the volume of 0.02 mol L^−1^ KMnO_4_ consumed of 2 g air-dried soil within 20 min (Li et al., 2014).

### 2.5 DNA extraction and high-throughput sequencing

For the extraction of soil macrogenomic DNA was the soil samples at three time points (June 15th, August 15th, and October 15th, respectively) in 2020 corresponding to shoot elongation, fruit enlargement, and early coloring maturity stage, which were the three peaks of nutritional and reproductive growth of table grapes.

The total genomic DNA in each sample (2.5 g field-moist soil) was extracted using hexadecyltrimethy ammonium bromide (CTAB) method (Zhou et al., 1996). DNA concentration and purity was monitored on 1% agarose gels. According to the concentration, DNA was diluted to 1 ng μL^−1^ using sterile water. The phusion^®^ High-Fidelity PCR Master Mix with GC Buffer (the New England Biological Laboratory Company) and the high-fidelity enzyme was used for the polymerase chain reaction (PCR). The V3–V4 region of the bacterial 16S rRNA gene was amplified using the PCR primers 515\u00B0F (5′-GTGCCAGCMGCCGCGGTAA-3′) and 806 R (50-GGACTACHVGGGTATCTAAT-30) (Guo et al., 2017), and the ITS2 region of fungi was performed using primers ITS3_KYO2F (50-GATGAAGAACGYAGYRAA-30) and ITS4-2409R (50-TCCTCCGCTTATTGATATGC-30) (Toju et al., 2012). The products from PCR amplification labeled with a barcode sequence were purified using GeneJET™ Gel Extraction Kit (Thermo Scientific) after electrophoresis in 2% agarose gel. Then, the 16S rRNA library was prepared by Ion Plus Fragment Library Kit 48 rxns (Thermo Scientific). Purified amplicons were sequenced on an Ion S5TM XL platform. Each sample data was separated from the off-line data according to the barcode sequence and PCR amplification primer sequence. After the barcode and primer sequence were cut off, the reads of each sample were spliced using FLASH (V1.2.7, http://ccb.jhu.edu/software/FLASH/) in order to obtain raw tags data (Magoč and Salzberg, 2011). Then, the high-quality tags data (clean tags) was obtained from strict filtering of raw tags using QIIME (V1.9.1, http://qiime.org/scripts/split_libraries_fastq.html) (Bokulich et al., 2013). After removing the chimeric sequence (http://www.drive5.com/usearch/manual/chimera_formation.html), compared with the database (Gold database, https://www.drive5.com/usearch/manual/chimera_formation.html) to detect the chimeric sequence, the final effective data (Effective Tags) was obtained. All effective tags of soil samples were clustered by Uparse software (Uparse v7.0.1001, http://drive5.com/uparse/). The sequences were clustered into OTUs (Operational Taxonomic Units) with 97% identity. At the same time, the sequence with the highest frequency in OTUs was selected as the COMMUNICATIONS IN SOIL SCIENCE AND PLANT ANALYSIS 5 representative sequence of OTUs, and the representative sequences of OTUs were annotated by using the Mothur method (http://www.arb-silva.de/) (Wang et al., 2007) and Silva’s SSUrRNA database (the threshold was set at 0.8 ~ 1) (Quast et al., 2013) to obtain taxonomic information of soil samples and make statistics on the community composition of each soil sample at each microbial community classification level.

### 2.6 Sequencing data processing

The multi-sequence alignment was performed using MUSCLE software (Version 3.8.31, http://www.drive5.com/muscle/) to obtain the phylogenetic relationship of all OTUs representative sequences (Edgar, 2004). Finally, the data of each sample was homogenized according to the standard of the least amount of data in the sample for the subsequent analysis of microbial community diversity. The alpha diversity values of grape rhizosphere soil bacteria, such as the species richness index (ACE, and Chao), and the species diversity index (Simpson, and Shannon) were calculated using the Qiime software (Version 1.9.1).

### 2.7 Statistical analysis

Statistical analyses were performed using Microsoft Excel 2019 software (Microsoft Corporation). The SPSS Statistics 26.0 software (IBM, Armonk, New York, United States) with the factorial analysis of variance (ANOVA) was applied for statistical validation and plotting was performed using Origin 2024 software (Origin Lab Corporation). The Duncan’s multiple range tests were undertaken for comparing all treatment means for significant difference. A redundancy analysis (RDA) was used to analyze the relationship between microbial community distribution and environmental explanatory variables, enzyme activities based on OTU level. Correlations among soil physicochemical properties, enzyme activities, and microbial biomass were assessed using Pearson correlation analysis.

## 3 Results

### 3.1 Effects of bacterial fertilizer application on greenhouse grape yield, water use efficiency and economic benefit under water stress conditions

**Figure 2:**
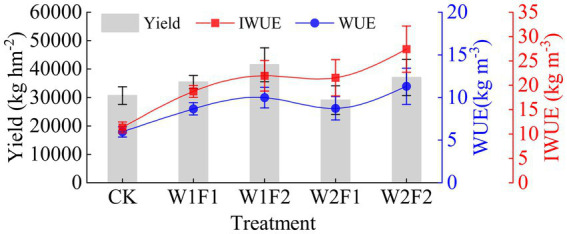
Greenhouse grape yield, water use efficiency (WUE) and irrigation water use efficiency (IWUE) in different treatments. CK, adequate water supply and no fertilization (soil moisture content was (75% ~ 100%) θ_f_); W1F1, mild water stress and small fertilizer (soil moisture content was (65% ~ 90%) θ_f_ and adding 60 kg hm^−2^ of bacterial fertilizer); W1F2, mild water stress and maximize fertilizer (soil moisture content was (65% ~ 90%) θ_f_ and adding 120 kg hm^−2^ of bacterial fertilizer); W2F1, moderate water stress and small fertilizer (soil moisture content was (55% ~ 80%) θ_f_ and adding 60 kg hm^−2^ of bacterial fertilizer); W2F2, moderate water stress and maximize fertilizer (soil moisture content was (55% ~ 80%) θ_f_ and adding 120 kg hm^−2^ of bacterial fertilizer).

The W1F2 treatment exhibited the highest yield (Figure 2), which was 35.44% higher and significantly higher (*p* < 0.05) than the CK treatment. No other treatments demonstrated a significant difference (*p* < 0.05) from the CK treatment (Table 2). At the same water stress gradient, both water use coefficient and irrigation water use coefficient increased with increasing fertilizer application. The water use coefficients of W1F2 and W2F2 treatments were significantly higher (*p* < 0.05) than that of CK control, but the differences with other treatments were not significant (*p* < 0.05); the irrigation water use coefficients of all treatments were significantly higher (*p* < 0.05) than that of CK treatment. Compared to CK treatment, W1F1, W1F2 and W2F2 treatments increased economic benefit (Equation (3)) by 19.31, 44.04, 25.31% (Table 2), respectively, but W2F1 treatment decreased by 7.21%. Compared with CK treatment, better economic benefits were W1F2 and W2F2 treatments.

**Table 2:** The influence of different water bacteria regulation on grape yield, water use efficiency and economic benefit.

Treatment	Yield (kg hm^−2^)	Irrigation quota (m^3^ hm^−2^)	Total water consumption (m^3^ hm^−2^)	Water use efficiency (WUE) (kg m^−3^)	Irrigation water use efficiency (IWUE) (kg m^−3^)	Basic input (CNY hm^−2^)	Fertilizer input (CNY hm^−2^)	Irrigation input (CNY hm^−2^)	Total input (TC) (CNY hm^−2^)	Total income (CNY hm^−2^)	Economic benefit (EB) (CNY hm^−2^)
CK	30644.72 ± 3075.59b	2,700	5135.44 ± 53.35a	5.97 ± 0.60b	11.35 ± 1.14e	37,480	22,488	1,027	60,995	275,796	214,801
W1F1	35416.67 ± 2279.91ab	1,890	4087.43 ± 96.93c	8.67 ± 0.73ab	18.74 ± 1.21 cd	37,480	24,168	817	62,465	318,750	256,285
W1F2	41505.56 ± 5978.49a	1,890	4157.35 ± 88.32c	9.98 ± 1.21a	21.96 ± 3.16abc	37,480	25,848	831	64,159	373,550	309,391
W2F1	29069.44 ± 5008.34b	1,350	3330.72 ± 77.18d	8.72 ± 1.37ab	21.53 ± 3.71abcd	37,480	24,168	666	62,314	261,625	199,311
W2F2	37016.67 ± 6369.27ab	1,350	3283.75 ± 157.25d	11.27 ± 2.12a	27.42 ± 4.72a	37,480	25,848	657	63,985	333,150	269,165

The data in tables denote the standard error of the mean (*n* = 3). Different letters in the same row mean significant difference at *p* ≤ 0.05 among the five treatments. CK, adequate water supply and no fertilization (soil moisture content was (75% ~ 100%) θ_f_); W1, mild water stress (soil moisture content was (65% ~ 90%)θ_f_); W2, moderate water stress (soil moisture content was (55% ~ 80%)θ_f_); W1F1, mild water stress and small fertilizer (soil moisture content was (65% ~ 90%) θ_f_ and adding 60 kg hm^−2^ of bacterial fertilizer); W1F2, mild water stress and maximize fertilizer (soil moisture content was (65% ~ 90%) θ_f_ and adding 120 kg hm^−2^ of bacterial fertilizer); W2F1, moderate water stress and small fertilizer (soil moisture content was (55% ~ 80%) θ_f_ and adding 60 kg hm^−2^ of bacterial fertilizer); W2F2, moderate water stress and maximize fertilizer (soil moisture content was (55% ~ 80%) θ_f_ and adding 120 kg hm^−2^ of bacterial fertilizer).

### 3.2 Soil physiochemical properties

**Figure 3:**
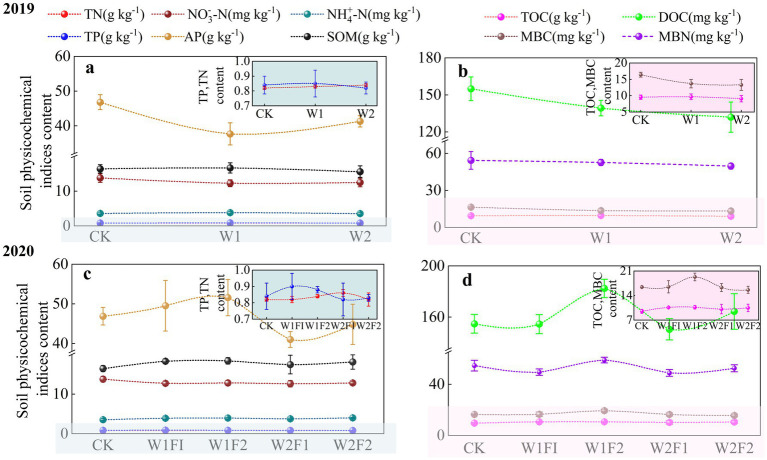
Effects of water stress and bacterial fertilizer addition on the TN, NO_3_-N, NH_4_-N, TP, AP, SOM **(A,C)**, and TOC, DOC, MBC, MBN **(B,D)** content of greenhouse grape rhizosphere soil in 2019 **(A,B)** and 2020 **(C,D)** at the coloring maturity stage. CK, adequate water supply and no fertilization (soil moisture content was (75% ~ 100%) θ_f_); W1, mild water stress (soil moisture content was (65% ~ 90%)θ_f_); W2, moderate water stress (soil moisture content was (55% ~ 80%)θ_f_); W1F1, mild water stress and small fertilizer (soil moisture content was (65% ~ 90%) θ_f_ and adding 60 kg hm^−2^ of bacterial fertilizer); W1F2, mild water stress and maximize fertilizer (soil moisture content was (65% ~ 90%) θ_f_ and adding 120 kg hm^−2^ of bacterial fertilizer); W2F1, moderate water stress and small fertilizer (soil moisture content was (55% ~ 80%) θ_f_ and adding 60 kg hm^−2^ of bacterial fertilizer); W2F2, moderate water stress and maximize fertilizer (soil moisture content was (55% ~ 80%) θ_f_ and adding 120 kg hm^−2^ of bacterial fertilizer). TN, total nitrogen; $NO_{3}^{-}-N$ , nitrate nitrogen; $NH_{4}^{+}-N$ , ammonia nitrogen; TP, total phosphorus; AP, availability phosphorus; SOM, soil organic matter; TOC, total organic carbon; DOC, dissolved organic carbon; MBC, microbial biomass carbon; MBN, microbial biomass nitrogen.

At the coloring maturity stage under water stress conditions in 2019 (Figures 3A,B), AP content decreased and then increased with increasing water stress gradient and was lower in both W1 and W2 treatments than in CK treatment, but the difference was not significant (*p* < 0.05) (Figure 3A), so there was a specific adaptation of AP content in the soil to water stress. While soil MBC, MBN and DOC contents were significantly lower (*p* < 0.05) in both W1 and W2 treatments compared with CK treatment (Figure 3B). It can be seen that water stress significantly suppressed soil MBC, MBN and DOC content, but had less effect on other physicochemical indexes.

Microbial fertilization additions significantly affected changes in rhizosphere soils AP, DOC, MBC and MBN (Figures 3C,D). Soil AP, DOC, MBC, and MBN contents were all highest under the W1F2 treatment, and all three indexes except AP were significantly higher than those of the CK treatment (*p* < 0.05). AP, MBC content among treatments showed W1F2 > W1F1 > CK > W2F2 > W2F1, indicating that the addition of bacterial fertilizer under mild stress could promote the accumulation of AP, MBC content, and the effect was more obvious with the increase of the amount of bacterial fertilizer. MBN, DOC contents showed W1F2 > CK > W2F2 > W1F1 > W2F1 among treatments, indicating that the increase in the amount of bacterial fertilizer promoted MBN, DOC contents more significantly under the same water stress gradient.

### 3.3 Soil enzyme activities

**Figure 4:**
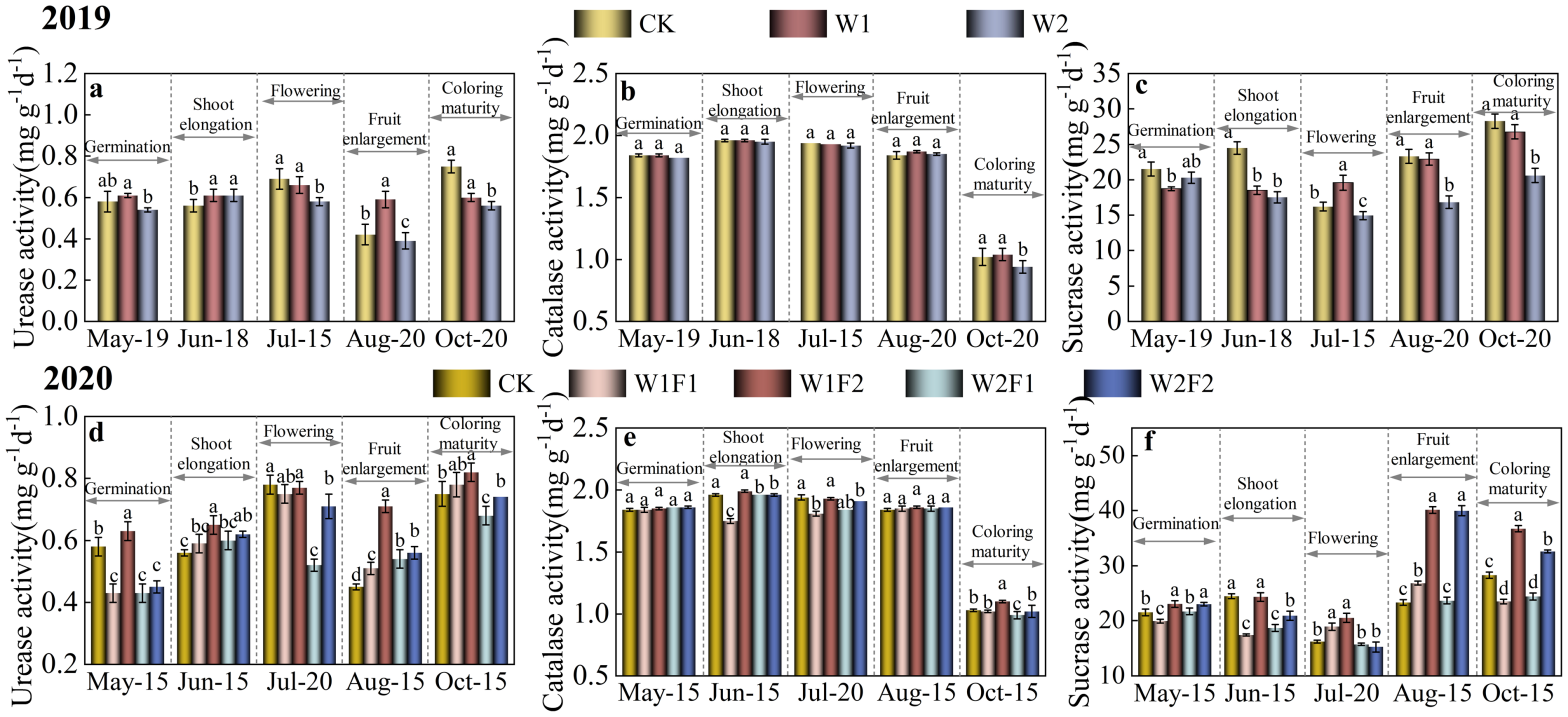
Effect of water stress and bacterial fertilizer addition on urease **(A,D)**, catalase **(B,E)**, and sucrase **(C,F)** enzyme activities in the rhizosphere soil of greenhouse grape in 2019 **(A–C)** and 2020 **(B–D)**, respectively. CK, adequate water supply and no fertilization (soil moisture content was (75% ~ 100%) θ_f_); W1, mild water stress (soil moisture content was (65% ~ 90%)θ_f_); W2, moderate water stress (soil moisture content was (55% ~ 80%)θ_f_); W1F1, mild water stress and small fertilizer (soil moisture content was (65% ~ 90%) θ_f_ and adding 60 kg hm^−2^ of bacterial fertilizer); W1F2, mild water stress and maximize fertilizer (soil moisture content was (65% ~ 90%) θ_f_ and adding 120 kg hm^−2^ of bacterial fertilizer); W2F1, moderate water stress and small fertilizer (soil moisture content was (55% ~ 80%) θ_f_ and adding 60 kg hm^−2^ of bacterial fertilizer); W2F2, moderate water stress and maximize fertilizer (soil moisture content was (55% ~ 80%) θ_f_ and adding 120 kg hm^−2^ of bacterial fertilizer).

Urease, catalase and sucrase activities were significantly lower (*p* < 0.05) in W2 treatment compared with CK during coloring maturity stage (Oct-15) (Figures 4A–C). In addition, the activities of various enzymes were slightly higher under the W1 treatment than under the W2 treatment from the germination stage (May-19) to the coloring maturity stage (October-15).

The changes in urease, catalase and sucrase activities were more pronounced with the addition of microbial fertilizer (Figures 4D–F). At the coloring maturity stage (Oct-15), all three enzyme activities were significantly higher (*p* < 0.05) in the W1F2 treatment than in the CK treatment. The W1F2 treatment increased soil urease activity at all fertility stages compared to the CK treatment and the differences were significant except at flowering stage.

### 3.4 Microbial richness and diversity

**Figure 5:**
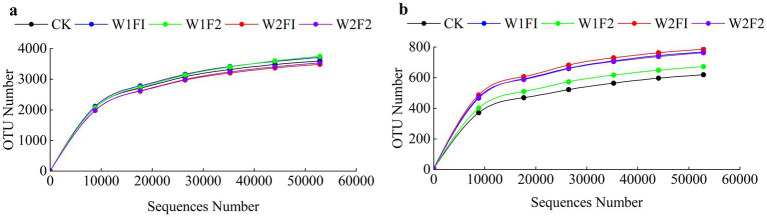
Rarefaction curve of each treatment of bacteria **(A)** and fungi **(B)** in the coloring mature period. CK, adequate water supply and no fertilization (soil moisture content was (75% ~ 100%) θ_f_); W1F1, mild water stress and small fertilizer (soil moisture content was (65% ~ 90%) θ_f_ and adding 60 kg hm^−2^ of bacterial fertilizer); W1F2, mild water stress and maximize fertilizer (soil moisture content was (65% ~ 90%) θ_f_ and adding 120 kg hm^−2^ of bacterial fertilizer); W2F1, moderate water stress and small fertilizer (soil moisture content was (55% ~ 80%) θ_f_ and adding 60 kg hm^−2^ of bacterial fertilizer); W2F2, moderate water stress and maximize fertilizer (soil moisture content was (55% ~ 80%) θ_f_ and adding 120 kg hm^−2^ of bacterial fertilizer).

Based on 16S rRNA and ITS gene sequencing analysis, we obtained a total of 1,201,770 and 1,183,320 effective tags from all 15 samples after the quality control. After OUT clustering, 8,754 and 2,028 OTUs were remained for bacterial and fungal community, respectively. The rarefaction curve analysis of three replicates mean sequences of each treatment in grapevine coloring maturity stage showed that the data volume of sequenced reads was reasonable and that increasing the number of reads made only a small contribution to the total number of OTUs, because all rarefaction curves approached the saturation plateau (Figure 5). The number of observed OTUs of all treatments in the coloring mature period was relatively stable. The diversity index of bacteria was calculated in Table 3. There was no significant difference (*p* < 0.05) in both of fungi and bacteria Simpson, Chao1, and Shannon at each treatment. Compared with CK, all treatments increased fungal Chao1 index. And the Shannon and Simpson index increased except W1F2 treatment. For bacterial diversity, compared with CK, the Shannon, Simpson and Chao1 index in all treatments decreased, except for Chao1 index, which was increased under mild water stress (W1F1 and W1F2 treatments). And under the same water condition, with the increase of bacterial fertilizer amount, Chao1 index increases, but Shannon index decreases in all treatments.

**Table 3:** Diversity of the 16S rRNA gene-based bacterial and ITS-based fungal communities in the coloring mature period in 2020.

Treatments	Bacteria			Fungi		
	Shannon	Simpson	Chao1	Shannon	Simpson	Chao1
CK	9.77 ± 0.25a	0.996 ± 0.00a	3,919 ± 222a	5.44 ± 1.14a	0.92 ± 0.06a	697 ± 133a
W1FI	9.72 ± 0.41a	0.995 ± 0.00a	4,081 ± 328a	5.91 ± 0.44a	0.94 ± 0.02a	833 ± 68a
W1F2	9.64 ± 0.41a	0.996 ± 0.00a	4,250 ± 793a	5.24 ± 0.47a	0.91 ± 0.03a	739 ± 31a
W2F1	9.52 ± 0.32a	0.995 ± 0.00a	3,828 ± 162a	6.33 ± 1.00a	0.96 ± 0.03a	854 ± 147a
W2F2	9.38 ± 0.10a	0.992 ± 0.00a	3,869 ± 273a	6.03 ± 0.76a	0.95 ± 0.02a	835 ± 18a

The data in tables denote the standard error of the mean (*n* = 3). Different letters in the same row mean significant difference at *p* ≤ 0.05 among the five treatments. CK, adequate water supply and no fertilization (soil moisture content was (75% ~ 100%) θ_f_); W1, mild water stress (soil moisture content was (65% ~ 90%)θ_f_); W2, moderate water stress (soil moisture content was (55% ~ 80%)θ_f_); W1F1, mild water stress and small fertilizer (soil moisture content was (65% ~ 90%) θ_f_ and adding 60 kg hm^−2^ of bacterial fertilizer); W1F2, mild water stress and maximize fertilizer (soil moisture content was (65% ~ 90%) θ_f_ and adding 120 kg hm^−2^ of bacterial fertilizer); W2F1, moderate water stress and small fertilizer (soil moisture content was (55% ~ 80%) θ_f_ and adding 60 kg hm^−2^ of bacterial fertilizer); W2F2, moderate water stress and maximize fertilizer (soil moisture content was (55% ~ 80%) θ_f_ and adding 120 kg hm^−2^ of bacterial fertilizer).

### 3.5 Bacterial and fungal community composition

**Figure 6:**
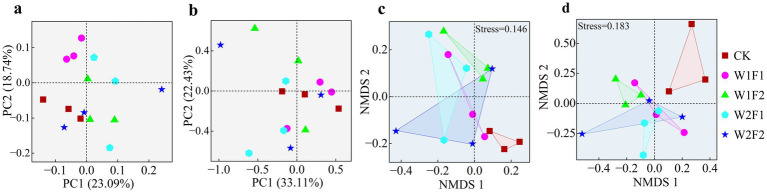
Principal co-ordinates analysis of bacterial **(A)** and fungal **(B)** based on weighted unifrac distance, and non-metric multi-dimensional scaling (NMDS) plots of bacteria **(C)** and fungi **(D)** based on bray-curtis distance.

Replicates from different treatments were largely clustered together indicating good sample reproducibility (Figure 6). The first axis of the PCoA analysis (Figures 6A,B) based on weighted unifrac distance for bacterial (a) and fungal (b) communities explained 23.09 and 33.11% of the variation, respectively. The bacterial communities were distinctly separated between the treatments with added bacterial fertilizer (as W1F1, W1F2 and W2F1) and CK, except for W2F2 (Figure 6A). The fungal communities however did not show a distinct separation among all treatments (Figure 6B). However, compared with CK treatment, soil bacterial and fungi communities were more heterogeneous with the addition of microbial fertilization, indicating variability in soil microbial communities. The NMDS (Figures 6C,D) based on bray-curtis distance revealed a clear separation among all treatments for both the 16S rRNA gene (c) and ITS gene (d) except W2F2 treatment, and showed that there were differences in the bacterial (*R* = 0.3333, *p* = 0.05) and fungal (*R* = 0.4763, *p* = 0.05) community structure between all treatments and CK according to Anosim test.

**Figure 7:**
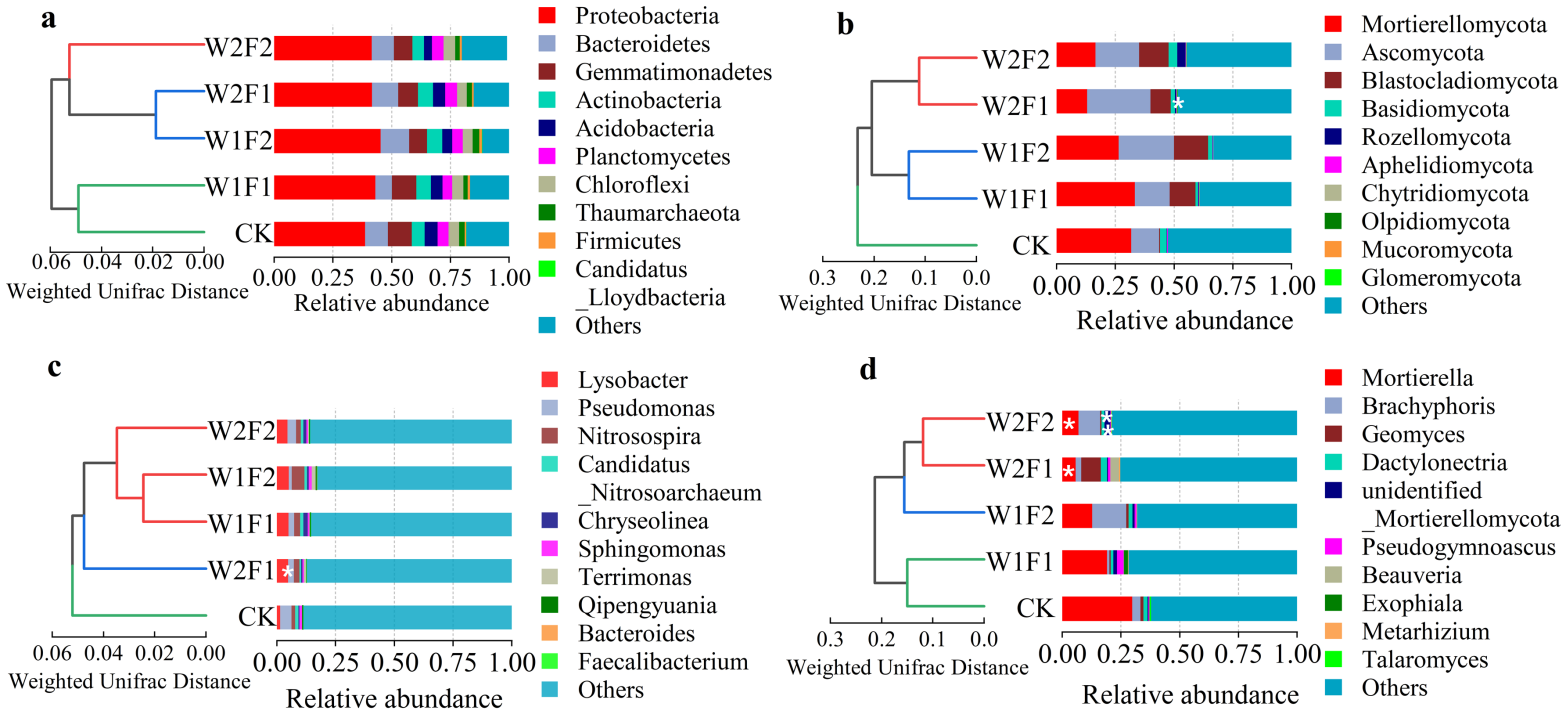
The top 10 16S rRNA gene-based bacterial [phylum **(A)** and genus **(C)**] and ITS gene-based fungal [phylum **(B)** and genus **(D)**] community compositions and UPGMA cluster analysis of bacteria [phylum **(A)** and genus **(C)**] and fungi [phylum **(B)** and genus **(D)**] based on weighted unifrac distance at the phylum and genus level in five treatments of coloring maturity stage in 2020. * and ** Indicates significant differences between each treatment and CK (* *p* < 0.05; ** *p* < 0.01).

The results of UPGMA (Unweighted Pair-group Method with Arithmetic Mean) cluster analysis of bacteria (phylum (a) and genus (c)) and fungi (phylum (b) and genus (d)) based on weighted unifrac distance in grape rhizosphere soil (Figure 7) showed that there were significant differences in the structure of bacteria and fungi in grape rhizosphere soil at phyla and genus levels under water stress and bacterial fertilizer addition.

Bacterial gene sequences were affiliated with 68 phyla and 568 genera. The dominant phylum in all soil samples was *Proteobacteria*, followed by *Bacteroidetes, Gemmatimonadetes, Actinobacteria, Acidobacteria, Planctomycetes, Chloroflexi, Thaumarchaeota, Firmicutes, Candidatus_Lloydbacteria,* representing average of 42.2, 9.9, 8.9, 5.9, 4.7, 4.6, 4.5, 2.3, 0.9, 0.02% of all sequences, respectively (Figure 7A). There were not significant differences in the relative abundances of all bacterial genes among the five treatments. However, for the top 10 phyla, W1F1, W1F2 and W2F1 treatments increased the relative abundance of *Actinobacteria* and the decrease was observed in W2F2 treatments, while that of *Thaumarchaeota* was significantly decreased by W1F1, W2F1 and W2F2 treatments, and increased in W1F2 treatment. Additionally, all treatments increased the relative abundance of *Proteobacteria* and *Firmicutes* and decreased of *Acidobacteria* compared with CK. At the genus level (Figure 7C), the top 10 genera in all soil samples were *Lysobacter, Pseudomonas, Nitrosospira, Candidatus_Nitrosoarchaeum, Chryseolinea, Sphingomonas, Terrimonas, Qipengyuania, Bacteroides, Faecalibacterium,* together representing an average of 13.1% of all sequences. That is, bacterial gene species were very rich at the generic level (Others accounted for 86.9%). When compared with CK, all treatments increased the relative abundances of *Lysobacter, Nitrosospira, Chryseolinea, Terrimonas,* while decreased that of *Pseudomonas* and *Faecalibacterium*, and significant difference (*p* < 0.05) in the relative abundances of *Pseudomonas* was observed between W2F1 treatment and CK.

Fungal gene sequences were affiliated with 11 phyla and 263 genera. The dominant phyla in all soil samples were *Mortierellomycota*, *Ascomycota*, *Blastocladiomycota*, *Basidiomycota*, *Rozellomycota*, *Aphelidiomycota*, *Chytridiomycota*, *Olpidiomycota*, *Mucoromycota*, *Glomeromycota*, together representing an average of 56.6% of all sequences (Figure 7B). There were significant differences (*p* < 0.05) in the abundance of *Glomeromycota* between W2F1 treatment and CK. All treatments significantly decreased the abundance of *Basidiomycota* and *Aphelidiomycota*, while increased that of *Ascomycota*, *Blastocladiomycota*, *Chytridiomycota*, *Olpidiomycota*, *Mucoromycota*, *Glomeromycota*. At the genus level (Figure 7D), the top 10 dominant genera were *Mortierella*, *Brachyphoris*, *Geomyces*, *Dactylonectr*, *unidentified_Mortierellomycota*, *Pseudogymnoascus*, *Beauveria*, *Exophiala*, *Metarhizium*, *Talaromyces*, representing approximately 28.9% of all sequences. There were significant differences (*p* < 0.05) in the abundances of *Mortierella* between W2F1, W2F2 treatments and CK, extremely significant differences (*p* < 0.01) in the abundances of *unidentified_Mortierellomycota* between W2F2 treatment and CK, and all treatments decreased the relative abundances of *Mortierella* compared with CK.

### 3.6 Relationships between the dominant genus of microbial communities and the characteristics of soil

**Figure 8:**
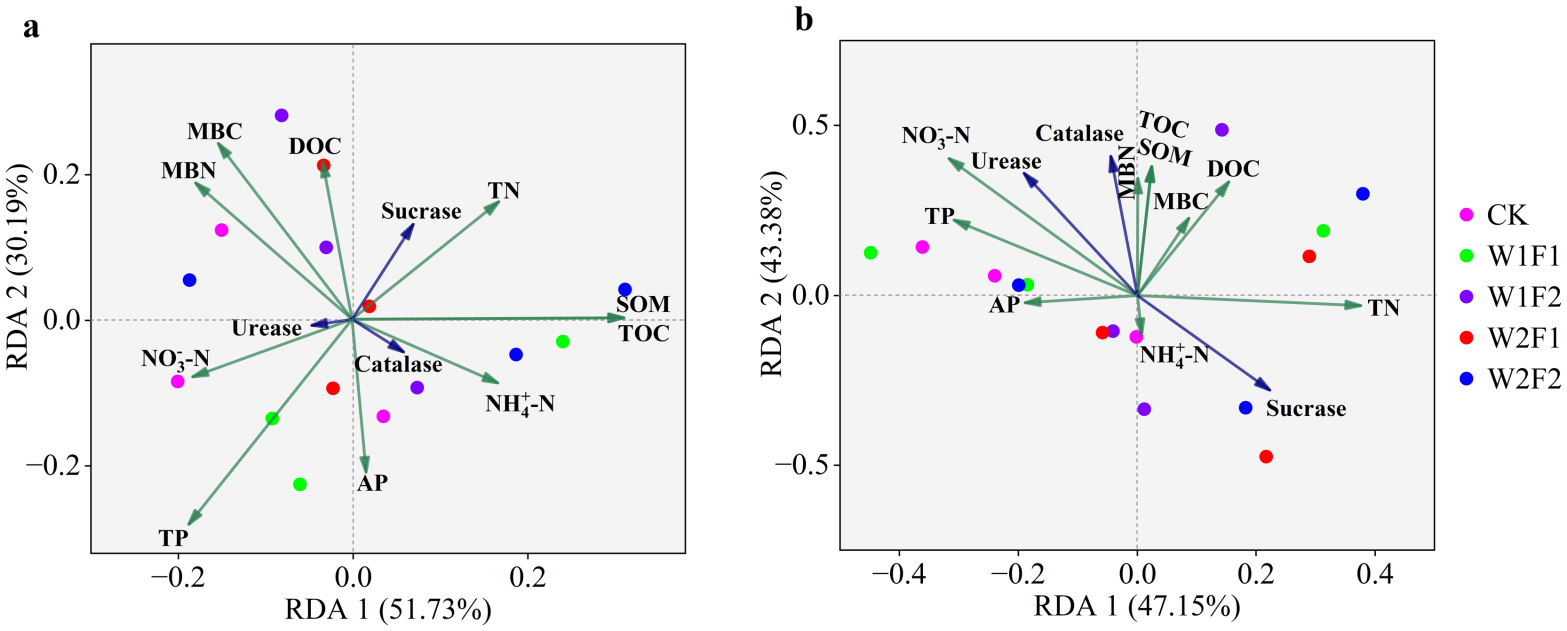
Redundancy analysis (RDA) of the bacterial **(A)** and fungal **(B)** communities and soil properties and enzyme activities for individual samples. Soil factors included total nitrogen (TN), total phosphorus (TP), available phosphorus (AP), nitrate nitrogen $(NO_{3}^{-}-N),$ ammonia nitrogen $(NH_{4}^{+}-N),$ total organic carbon (TOC), dissolved organic carbon (DOC), soil organic matter (SOM), microbial biomass nitrogen (MBN), microbial biomass carbon (MBC), urease, sucrase and catalase activities. The direction of the arrows indicates correlations with the first two canonical axes, and the length of the arrows represents the strength of the correlations.

**Figure 9:**
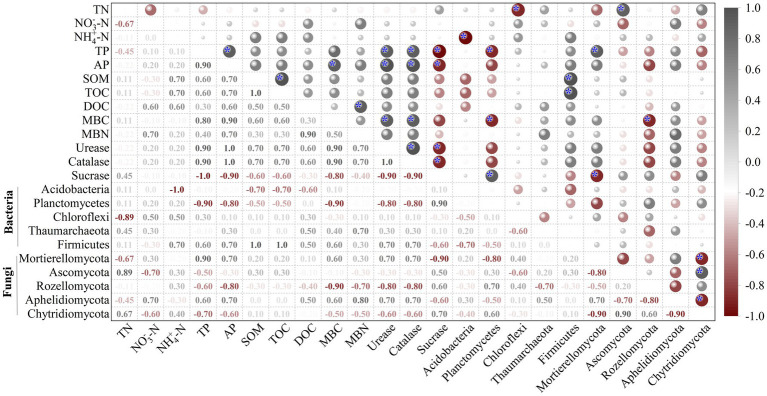
Correlation plot reveals the relationships between soil properties and the bacterial and fungal abundant phylum with significant differences in the rhizosphere soils under four fertilization regimes. *, *p* < 0.05.

Redundancy analysis (RDA) showed that SOM and TOC were key driving factors for the bacterial communities in the rhizosphere soils (Figure 8A), while TP, MBC, and MBN were the highly associated factors. For fungi, NO_3_^−^-N and TN were key driving factors (Figure 8B). The correlations (Spearman) heatmap results were shown in Figure 9. In addition to the negative correlation between NO_3_^-_^N and TN, SOM, TOC, between TN and TP, AP, positive correlations were found between all of other soil physicochemical indicators. Among them, AP was significantly positively correlated with TP, MBC (*p* < 0.05); SOM was significantly positively correlated with TOC (*p* < 0.05); and MBN was positively correlated with DOC (*p* < 0.05). The correlation analysis between soil physicochemical indicators and soil enzyme activities showed that there were positive correlations between soil urease, catalase and physicochemical indicators to varying degrees, while there was a negative correlation between sucrase and soil physicochemical indicators. Among them, urease and catalase were significantly positively correlated with MBC, AP and TP (*p* < 0.05); sucrase was significantly negatively correlated with TP and AP (*p* < 0.05). Furthermore, the *Acidobacteria* abundance correlated negatively (*p* < 0.05) with NH_4_^+^-N, the *Chloroflexi* abundance correlated negatively (*p* < 0.05) with TN, and the *Planctomycetes* abundance correlated negatively (*p* < 0.05) with MBC and TP, while the *Firmicutes* abundance correlated positively (*p* < 0.05) with TOC and SOM. For fungi, the *Ascomycota* abundance correlated positively (*p* < 0.05) with TN, the *Mortierellomycota* abundance correlated positively (*p* < 0.05) with TP, while the *Rozellomycota* abundance correlated negatively (*p* < 0.05) with MBC. There was a correlation between soil enzyme activities and the relative abundance of bacteria and fungi, with sucrase being positively (*p* < 0.05) correlated with the *Planctomycetes* abundance and negatively (*p* < 0.05) correlated with the *Mortierella* abundance. It is worth noting that urease and catalase had little effects on *Acidobacteria*.

## 4 Discussion

In arid and semi-arid region, limited water resources can lead to significant reductions in crop productivity and fruit quality. The lack of soil water directly affects the availability of nutrients in contact with the plant root system and the overall water balance in the plant, leading to a decrease in cellular tension, causing physiological imbalances in the crop, which in turn limits crop growth (Chaves et al., 2009). Studies have shown that the addition of microorganisms associated with the plant root system represents a sustainable strategy to mitigate the effects of drought stress on crop growth and yield (Lamaizi et al., 2023). In this study, we investigated the effects of bacterial fertilizer application, as a sustainable approach, on the rhizosphere soil environment and grape yield under water stress conditions.

Soil nutrients are comprehensive reflection of the physicochemical and biological properties of the soil, which can directly affect soil productivity. They are the basic source of nutrients required for the growth of various crops, which are of great importance within the soil ecosystem. The results showed stronger selective absorption of NO_3_^−^-N compared with NH_4_^+^-N, as well as the promotion of the absorption of AP content in the soil under water stress (Figures 3A,B). Of note, the effects of three water gradient treatments on nitrogen-related indicators (TN, NH_4_^+^-N and NO_3_^−^-N) and TP contents were not significant. The results were consistent with previous findings that soil TN and TP contents were insensitive indicators under water stress (Zhang et al., 2024). In addition, the study also found that water stress did not significantly affect rhizosphere soil TOC content during coloring maturity stage (Oct-15). A possible explanation may be that grape rhizosphere exudates were first made available to rhizosphere soil microorganisms for utilisation and colonization (Song et al., 2012), while the accumulation of soil organic carbon pool reserves was a long process, and changes in grapevine root exudates volume induced by short-term water stress (one growth cycle) were not sufficient to cause significant changes in soil organic carbon content. Meanwhile, the quality of SOM was maintained under the water deficit condition (Wu et al., 2011), but soil DOC showed a trend of decreasing with the increase of drought stress intensity. This finding was contrary to previous results that suggested DOC could increase with the decline of soil moisture for drought reduced the leaching of DOC from the soil and decreased the microbial activity associated with DOC consumption (Wang et al., 2017). In principal, DOC in soils results from microbial activity, root exudation or leaching from litter and humus, i.e., DOC is a transient stage in the decomposition of SOM (Hagedorn and Joos, 2014). However, the specific sources of DOC in soil vary by ecosystem and soil type, and the relative weights of the various sources remain uncertain (Kaiser and Guggenberger, 2000). The results of the present study showed that water stress significantly suppressed MBC, MBN and DOC content in soil (Schimel et al., 1999), this could be explained by the fact that water stress reduced the vitality of plant roots, which in turn caused the reduction of various organic and inorganic substances secreted by plant roots. In conclusion, with the exception of AP and DOC, water stress had less effect on soil physicochemical properties. The addition of bacterial fertilizer could effectively change the physicochemical properties of soil under water stress. In this study, we found that the W1F2 treatment had the largest of the four indexes of soil AP content, DOC, MBC and MBN content, and the other three indexes except AP content were significantly higher than that of the control CK. At the same application rate of bacterial fertilizer, the AP content, DOC content, MBC content and MBN content of the soil decreased with the increase of the water stress gradient; however, they also increased with the increase of bacterial fertilizer application rate under the same water stress gradient; especially under mild water stress, the treatment groups with different amounts of microbial fertilizer applied were larger than the CK treatments in terms of soil AP and SOM contents, indicating that soil organic matter conditions were improved after microbial fertilizer application, and P fixation was reduced, thereby improving soil P availability. This may be due to the fact that microbial fertilizers contain a wide range of microorganisms and mild water stress favours the enhancement of their activity, which in turn increases the SOM content and hence soil fertility (Liu et al., 2022).

Soil enzymes are significant driving force in the metabolic process of soil ecosystems. Their activities reflected the exuberance of soil material and energy metabolism and served as an important indicator for evaluating soil fertility and ecological environment quality. This study showed that at all reproductive stages of greenhouse grape growth, water stress inhibited the activities of urease, catalase and sucrase in soil, and the inhibition gradually increased with prolonged stress time and increasing stress level. The results were consistent with previous finding that soil enzyme activities were greatly inhibited by drought (Sardans and Peñuelas, 2010). By the coloring maturity stage, the activities of the three types of enzymes in treatment W1 were lower than those in CK, but the differences were not significant, and those in treatment W2 were all significantly lower (*p* < 0.05) than CK. The principal reason may be attributed to the reduction in soil water availability, which caused changes in the nutrient quality of the enzyme substrate, while substrate diffusion and limited oxygen content made the rhizosphere soil conditions anaerobic microcosms and thus inhibited the enzyme activity (Sardans and Peñuelas, 2010). Another reason may be that water stress alters soil microbial populations and species by means of osmotic stress and resource competition (Chodak et al., 2015), since the enzymatic activity of soil peroxidase was related to the number and species of microorganisms in the soil. Reduced activities of enzymes such as proteases, ureases and deaminases had been reported due to toxic effects caused by trace elements present in organic amendments (Hargreaves et al., 2008). Furthermore, the activities of urease and acid phosphatase in the mesotrione-treated and control soil were not different from 2nd to 20th day after application (Du et al., 2018). The silver nanoparticles (AgNPs) had minor influence on the soil physicochemical properties and enzyme activities (de Oca-Vásquez et al., 2020). Meanwhile, the application of novel bioorganic fertilizer (BIO) did not change the main soil chemical properties and enzyme activity in China rice paddies (Li et al., 2021). In contrast, diverse studies had shown that microbial fertilizer application was associated with an increase in soil enzyme activities due to the beneficial secretions of active bacterial fertilizer (Martínez et al., 2018; Ekici et al., 2023). The present study showed that significant changes in urease, catalase and sucrase activities were observed with the addition of bacterial fertilizer under water stress. In particular, the activities of all three enzymes were significantly higher (*p* < 0.05) in W1F2 treatment than CK when water stress persisted until the coloring maturity period (October to 15). Among the enzymes selected in this study, urease and catalase are associated with the transformation of carbon, nitrogen and phosphorus in soil (Dick, 1994). Furthermore, soil microbial biomass was positively correlated with soil enzyme activities (Acosta-Martínez and Harmel, 2006). Therefore, the addition of maximize bacterial fertilizer under mild water stress conditions (W1F2) may be effective in improving the rhizosphere soil nutrient environment of greenhouse grape by promoting beneficial soil enzyme activities.

The W2F1 and W2F2 treatments were observed to reduce bacterial diversity, whereas all fertilizer addition treatments demonstrated an increase in fungal diversity. This suggested that the introduction of bacterial fertilizer under conditions of mild water stress facilitates an enhancement in microbial diversity in rhizosphere soil of greenhouse grape. In the present study, the dominant phylum was *Proteobacteria*, which constituted the largest bacterial group. This phylum encompasses numerous nitrogen-fixing bacteria, such as *Rhizobium* (Spain et al., 2009). *Lysobacter*, a kind of Gram-negative bacteria, is the most important dominant bacterial genus, with unique lysogenic activity, rapid reproduction, convenient storage, environmentally friendly, etc. It not only produces abundant extracellular enzymes to dissolve the cell walls of bacteria, fungi, nematodes, oomycetes, cyanobacteria and other pathogens for inhibiting other bacteria (Chen et al., 2016), but also synthesises a variety of secondary metabolites with broad-spectrum antimicrobial activity for regulating plant diseases (Yang et al., 2024), which may be the reason why this bacterium can be the dominant bacterium in the rhizosphere soil of greenhouse grape. In our study, all fertilizer treatments increased the abundance of *Lysobacter*, that is, bacterial fertilizer application under water stress conditions may be a viable option to improve the soil environment (e.g., salinisation) of greenhouse grape in arid regions.

Furthermore, the application of fertilizer has been demonstrated to influence the composition of fungal communities. The present study revealed that the dominant fungal phylum was *Mortierellomycota*, which contrasts with previous research indicating that nearly all fungi isolated from agricultural fields belonged to the *Ascomycetes* phylum (Viaud et al., 2000). A significant reduction in the relative abundance of *Mortierellomycota* was observed in all fertilizer treatments when compared with the CK treatment. Conversely, a significant increase in the relative abundance of *Ascomycetes*, which ranked second in abundance, was noted. *Mortierellomycota* were important in ecology, and although their specific ecological functions had been less studied, their role in soil microbial communities could not be ignored. For example, in cedar soils, *Mortierellomycota* was one of the most dominant taxa, accounting for about 76.71–86.72% of the total fungal community together with two other taxa. *Modicella*, a new species of sporocarp-forming fungus from New Zealand (*Mortierellaceae: Mortierellomycota*), which is an unusual genus within the *Mortierellaceae* because it forms relatively large terrestrial sporocarps, was the dominate dominant fungal genus, and its role in the rhizosphere soil microbiological environment of greenhouse grape need to be further investigated.

Previous studies have shown that environmental factors such as pH, SOC, available phosphorus and enzyme activities determine soil microbial communities in different ecosystems (Chen et al., 2019). In this study, RDA analysis and Correlation plot showed that TOC, SOM, TN, TP, MBC, and sucrase were the major determinants of the total bacterial community composition when microbial fertilizer were applied under water stress conditions. Unlike bacteria, fungal community composition was significantly related to NO_3_^−^-N, TN, and sucrase. These results suggested that soil properties and enzyme activities were the driving factors of microbial community composition when microbial fertilizer were applied under water stress conditions. It remains to be further investigated whether the application of bacterial fertilizers under water deficit conditions mitigates the adverse effects of water stress by providing nitrogen, producing growth promoters, increasing microbial populations, improving nutrient availability and uptake, and thereby influencing plant physiological properties, such as promoting leaf chlorophyll synthesis.

Our experiments were conducted in greenhouse, there were certain limitations, and whether the findings are equally applicable to field grapes needs to be further investigated. The present study focused on soil physicochemical properties, enzyme activities, and microbial composition. However, the causal relationship between these changes and bacterial fertilizer application remains to be elucidated, which is the main direction of our later research. We hypothesized that analyzing an analysis of plant root exudates, soil nutrient transport processes within the plant, and plant photosynthesis in conjunction with the current findings would better explore the intrinsic mechanisms by which bacterial fertilizer alleviate water stress and improve the soil environment from the physiological and biochemical perspectives of the grapevine. In addition, it usually takes several years for biological, chemical and physical processes in the soil to show significant changes. Therefore, long-term monitoring of these parameters should be considered in future studies.

## 5 Conclusion

The study revealed notable differences in rhizosphere soil enzyme activities and microbial biomass of greenhouse grape plants subjected to water stress (*p* < 0.05), while the impact of water stress on soil physicochemical properties was relatively limited. The addition of bacterial fertilizer under water stress conditions significantly increased AP, DOC, MBC and MBN contents in the rhizosphere soil of greenhouse grape. Urease, catalase and sucrase activities were significantly higher in the W1F2 treatment than in CK (*p* < 0.05). Furthermore, bacterial fertilizer treatments altered both bacterial (16S rRNA gene) and fungal (ITS gene) diversity. While all fertilizer treatments increased fungal diversity, W2F1 and W2F2 treatments decreased the bacterial diversity, whereas W1F1 and W1F2 treatments showed the opposite effect. The bacterial fertilizer significantly affected the overall bacterial and fungal community structures and composition. The dominant bacterial phylum and genus were *Proteobacteria* and *Lysobacter*, while the dominant fungal phylum and genus were *Mortierellomycota* and *Mortierella*, respectively. Soil TOC, SOM, TN, TP, MBC, and sucrase were identified as key environmental factors shaping the bacterial community structure, whereas the fungal community was mainly influenced by NO_3_^−^-N, TN, and sucrase. These results indicate that bacterial fertilizer application under water stress conditions significantly affected soil C, N, and P cycling. Regarding grape yield and water use efficiency, only the W1F2 treatment showed significantly higher values compared to the control (CK), while other fertilizer treatments showed no significant differences, and the W1F2 treatment was also the most economically efficient. Overall, the application of appropriate amounts of microbial fertilizer under water stress conditions can improve grape yield by enhancing the rhizosphere soil environment and soil fertility of greenhouse grape, thereby increasing the economic income of farmers. To achieve maximum economic benefits, mild water stress combined with maximum microbial fertilization (W1F2) is recommended as the optimal water and fertilizer management strategy for greenhouse grape in the arid and semi-arid region of Northwest China.

## Data Availability

The data presented in the study are deposited in the NCBI Sequence Read Archive (SRA) database, accession number PRJNA1214986.

## Footnotes

^1^ www.ruichubio.com
